# Program Evaluation of Community Case Management with Reactive Test and Treat for Malaria in a High-Transmission Setting

**DOI:** 10.4269/ajtmh.24-0405

**Published:** 2025-04-22

**Authors:** Austin Weynand, Manuela Hauser, Caitlin Bond, James Sichivula Lupiya, Dickson Phiri, Bruce Phiri, Molly Mantus, Benjamin Kussin-Shoptaw, Mike Chaponda, Mbanga Muleba, Jean-Bertin B. Kabuya, Gershom Chongwe, William J. Moss, Matthew M. Ippolito

**Affiliations:** ^1^ University of Texas Medical Branch, Galveston, Texas;; ^2^ Department of Paediatrics, Cantonal Hospital Graubuenden, Chur, Switzerland;; ^3^ Department of International Health, Johns Hopkins Bloomberg School of Public Health, Baltimore, Maryland;; ^4^ Tropical Diseases Research Centre, Ndola, Zambia;; ^5^ Saint Paul’s General Hospital, Luapula, Zambia;; ^6^ Johns Hopkins Bloomberg School of Public Health, Baltimore, Maryland;; ^7^ Department of Epidemiology, Johns Hopkins Bloomberg School of Public Health, Baltimore, Maryland;; ^8^ Malaria Research Institute, Johns Hopkins Bloomberg School of Public Health, Baltimore, Maryland;; ^9^ Department of Medicine, Johns Hopkins School of Medicine, Baltimore, Maryland

## Abstract

Community case management (CCM) combined with reactive test-and-treat (RTAT) for malaria was implemented by the National Malaria Elimination Program in a holoendemic region of Zambia. We assessed the impact of CCM + RTAT activities on malaria care seeking, health facility cases, and hospital mortality. We analyzed data from community surveys, a health facility-based passive surveillance network, and a hospital-based severe malaria surveillance system to compare metrics across the program eras (July 2016–July 2018, August 2018–October 2019, and November 2019–July 2021). Geospatial mapping was used to visualize trends in referrals and mortality. Clinical profiles of 696 hospitalized children with malaria were compared and in-hospital mortality were analyzed across periods using multiple logistic regression. There were more frequent health contacts for malaria reported by community members and a corresponding decrease in health facility malaria cases during CCM + RTAT. Pediatric patients admitted to the hospital with malaria during CCM + RTAT had less severe disease and shorter lengths of stay and in-hospital mortality was lower (odds ratio: 0.24, 95% CI: 0.07–0.84, *P* = 0.025). Geospatial mapping of the home villages of children hospitalized with malaria showed a wider catchment during CCM + RTAT than before or after. In this high malaria transmission setting, CCM + RTAT increased access to care, shifted malaria case burden from health facilities to community health workers, and improved in-hospital outcomes for malaria, likely from earlier referral. However, RTAT + CCM in this high-transmission area proved unsustainable because of excessive consumption of malaria commodities.

## INTRODUCTION

Malaria is the leading cause of pediatric morbidity and mortality in areas of Africa where it is holoendemic. For nearly a decade, few inroads have been made in malaria control in many of these settings. Global malaria case incidence and mortality have been essentially constant since 2015, and in some areas, malaria has resurged.^1^ Public health initiatives that lessen its toll are urgently needed.

Community case management (CCM) aims to provide early, effective treatment to children with malaria in areas where access to health facilities is limited.^2^ By training community health workers (CHWs) and equipping them with antimalarial medications and rapid diagnostic tests, CCM seeks to bring essential health services closer to communities. The intent is to reduce the burden on health facilities and to increase early diagnosis, treatment, and referral to reduce disease progression and death.

Reactive test and treat (RTAT) is a malaria control strategy in which individuals residing in proximity to a confirmed malaria case are tested using a rapid diagnostic test and, if positive, treated.^3^ RTAT aims to quickly identify and treat secondary cases to reduce transmission. Conventionally, RTAT is reserved for areas of low malaria transmission, where the number of cases is manageable and the amount of commodities needed to sustain the program (e.g., diagnostic tests, medication, and fuel) are available and affordable. It is generally inadvisable for RTAT to be implemented in areas of high malaria transmission.^3^

In Africa, CCM has had variable success, with some programs achieving reductions in malaria mortality and others demonstrating CHWs’ ability to accurately diagnose, treat, and refer.^2^^,^^4^^–^^6^ RTAT is included in the guidelines of several African control programs, including those of Zambia, as a tool for elimination in areas with low malaria incidence.^7^

With support from the U.S. President’s Malaria Initiative, the Zambian National Malaria Elimination Program implemented malaria CCM in the high-transmission district of Nchelenge, informed in part by our prior work that identified distance from the hospital as the strongest risk factor for in-hospital malaria mortality.^8^ In 2018, 385 CHWs were trained and equipped for CCM according to a malaria operational plan developed by the Zambia Ministry of Health.^9^ A decision was made by ministry officials to also have the CHWs conduct RTAT. Both CCM and RTAT were programmatic interventions and not research activities.

The combined CCM and RTAT (CCM + RTAT) activities took place over 15 months from September 2018 to November 2019 until commodities were exhausted and both CCM and RTAT activities were paused. The timeline of events constituted a natural crossover design for evaluating CCM + RTAT by comparing the pre-, intra-, and postprogram periods.

To study the impact of the program, we used data collected through ongoing epidemiological studies of malaria in the area that included a community cohort, passive health facility surveillance, and sentinel surveillance for severe malaria at the district hospital. We also leveraged weather and entomological data to account for background trends that could have influenced the analysis. We hypothesized that CCM + RTAT would be associated with increased health care contacts for malaria, a shift in malaria case burden from health facilities to CHWs, and an increase in early referrals, which would be reflected in the hospital data as decreased severity of malaria and reduced mortality. Pediatric hospitalizations for malaria might be expected to increase as more severe cases are detected, decrease if early treatment effectively prevents mild cases from worsening, or remain stable depending on the balance of the two.

## MATERIALS AND METHODS

### Study site and population.

The study was conducted in Nchelenge District, Luapula Province in northern Zambia. The district is among the highest burden areas and records the highest malaria-associated mortality in the country.^10^ A single district hospital serves a population of 242,000 residents, including 55,100 children younger than 5 years old. Health facility cases of malaria ranged from 158 to 340 per 1,000 population per year, with half of cases occurring in children younger than 5 years old.^11^ Hospitalizations for malaria ranged from three to seven per 1,000 population per year, with children younger than 5 years old accounting for 60%.^11^ Vector control with annual indoor residual spraying and periodic bed net distributions has been implemented in the study area with high coverage but minimal impact on parasite prevalence.^11^

### Intervention.

**Figure 1.**
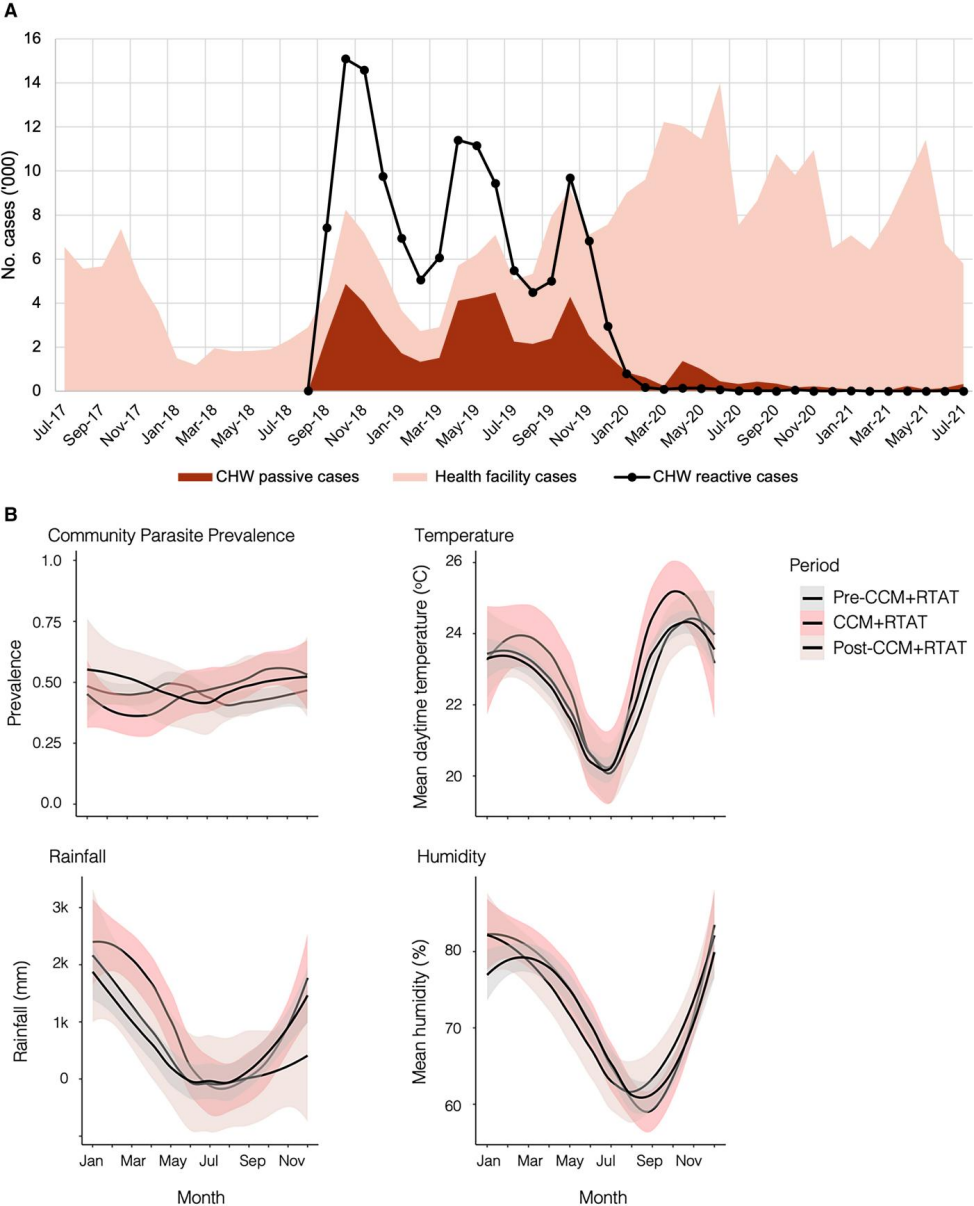
(**A**) Time series of community health worker (CHW)- and health facility-confirmed malaria cases are displayed as overlying plots, and CHW reactively detected cases are displayed as an overlaid line graph. Data were provided by the National Malarial Elimination Program. (**B**) Trends in weather and background parasite prevalence were measured in the community survey participants by rapid diagnostic test across the three periods. CCM = community case management; RTAT = reactive test-and-treat.

CHWs were trained by the program to diagnose, triage, treat, and refer children with malaria. CHWs were supplied with rapid diagnostic tests and artemisinin-based combination therapy (artemether-lumefantrine as Coartem™ manufactured by Novartis AG, Basel, Switzerland). The ministry instructed the CHWs to conduct RTAT, whereby CHWs visited the homes and surrounding homes within a 140-m radius of passively detected malaria cases to screen for malaria using a rapid diagnostic test and treat occupants who tested positive. The CHWs were active for a 15-month period from September 2018 to November 2019 (Figure 1). After this time, owing to commodity stock outs, they effectively ceased their activity.

### Data collection.

Longitudinal and cross-sectional household surveys were conducted monthly as described previously.^11^ Briefly, households (*n* = 1,874) were randomly selected for inclusion from satellite images. All household members (*N* = 7,732) were screened using a rapid diagnostic test (SD Bioline, Gyeonggi-do, Republic of Korea) and administered a survey that included health care utilization assessments, although the survey instrument did not differentiate between facility-based and CHW encounters for malaria care. Individuals who tested positive were offered treatment with artemether-lumefantrine. Temperature and rainfall data were collected through a locally installed weather station (HOBO, Onset Computer Corporation, Bourne, MA). Weekly confirmed malaria cases were reported from the 15 health facilities within the hospital catchment area. Aggregate and individual-level data on hospitalized children 15 years old and younger with malaria were collected from hospital registers and laboratory records as previously described, including home village.^12^ Missing leukocyte and platelet data were multiply imputed using a fully conditional specification with 25 iterations.^13^ To reduce misclassification bias,^14^ the analysis of hospital patients was restricted to those who met platelet and leukocyte thresholds for severe malaria defined by Watson et al.^15^ (*n* = 696). Community survey participants provided written informed consent, and study research plans were approved by the Johns Hopkins Bloomberg School of Public Health Institutional Review Board and the Tropical Diseases Research Centre Ethics Review Committee in Zambia.

## STATISTICAL ANALYSES

Data were stratified by time period: preprogram (July 2016–July 2018), intraprogram (August 2018–October 2019), and postprogram (November 2019–July 2021). Household member data on malaria care-seeking indicators were compared using generalized estimating equations clustered on household and adjusted for age, sex, bed net use, and indoor residual spraying. Health facility data were analyzed in Poisson regression models conditioned on month of year. Individual patient data were analyzed by multiple logistic regression of mortality on CCM + RTAT period adjusted for age, sex, hemoglobin, distance of home village to hospital, and codiagnosis of gastroenteritis, which was found to be independently associated with mortality. Subgroup analyses were done by age, sex, and distance to the hospital. Community parasite prevalence by rapid diagnostic test was analyzed using the χ^2^ test, and temperature, rainfall, and humidity were graphed according to calendar month and tested using one-way analysis of variance. Characteristics of hospitalized children with severe malaria were compared across CCM + RTAT periods using the Kruskal–Wallis test for continuous measures and the χ^2^ or Fisher exact test for categorical measures. Coordinates of villages and CHW locations were collected and mapped as previously described.^16^ Health care access was visualized using inverse distance weighted interpolation of 20 village clusters, where each cluster was assigned a value equal to the proportion of hospitalized patients with malaria originating from that cluster in a given period. Statistical analysis was done using Stata 18 (StataCorp, College Station, TX).

## RESULTS

### Background characteristics.

Background parasite prevalence, measured as the proportion of community participants with a positive rapid diagnostic test, was similar across the periods but slightly higher in the post-CCM + RTAT period (46% versus 46% versus 51%, *P* <0.001) (Figure 1). Bed net usage was similar (72% versus 70% versus 72%, *P* = 0.36) and indoor residual spraying was lower in the pre-CCM + RTAT period (8% versus 23% versus 19%, *P* <0.001). Temperature, humidity, and rainfall were similar across the three periods (Figure 1).

### Malaria case burden and health system engagement.

**Figure 2.**
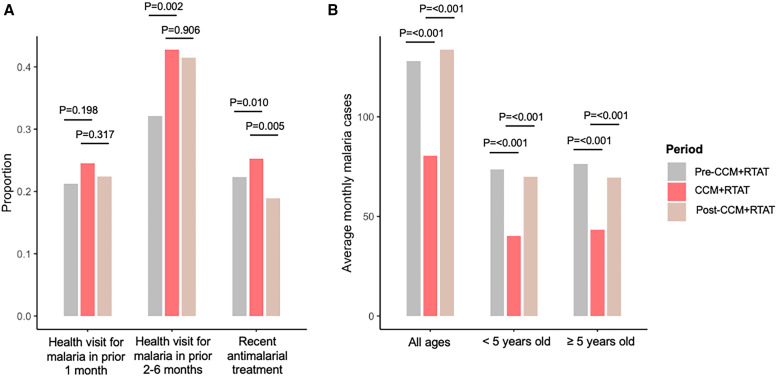
Community and health facility surveillance data showed a shift in health care utilization for malaria during the community case management (CCM) + reactive test-and-treat (RTAT) period. (**A**) Reported health provider visits and antimalarial treatment by period. Comparisons show *P*-values from generalized estimating equations clustered on household and adjusted for age, sex, bed net use, and indoor residual spraying (*N* = 7,732 study participants from 1,874 households). (**B**) Average monthly malaria cases presenting to local health facilities stratified by age group and period. Comparisons show *P*-values from Poisson regression adjusted for month of year.

Total detected malaria cases increased from a baseline average of 3,513 per month before CCM + RTAT to 13,544 cases per month during the intervention period when accounting for health facility cases, CHW passive cases, and CHW reactive cases (Figure 1). After CCM + RTAT ended, average monthly cases decreased to 9,687, reflecting declines in both CHW passive cases (from 2,843 to 535 per month) and reactive case detection (from 8,115 to 540 per month), whereas health facility cases increased (2,587 versus 8,611). More community participants reported health system contact for malaria (CHW and/or health facility) in the CCM + RTAT period compared with before and after CCM + RTAT, whereas there was a significant decrease (−38%, *P* <0.001) in malaria cases reported by health facilities that quickly returned to pre-CCM + RTAT levels once CHWs were inactive (Figure 2). Health facilities saw on average 2.7 fewer cases per week during CCM + RTAT for each CHW who was affiliated with it (95% CI: −2.3 to −3.2, *P* <0.001, Poisson regression adjusted for month of year). RTAT detected and treated 128,592 additional rapid diagnostic test-positive cases.

### Hospitalizations for malaria.

**Figure 3.**
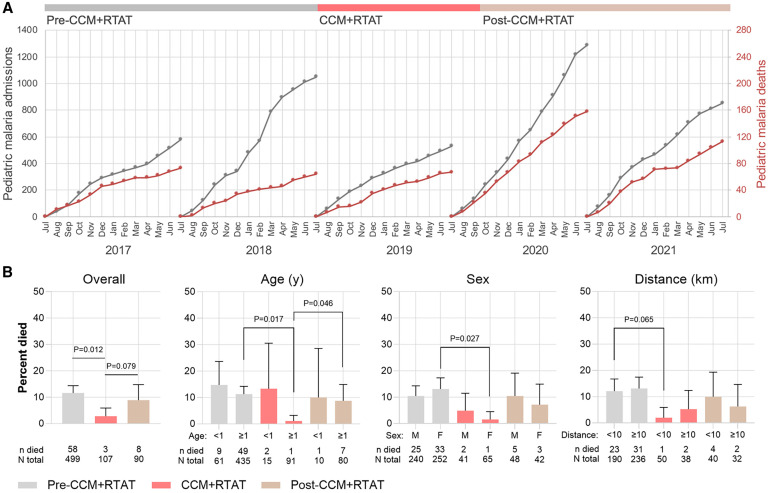
(**A**) Annual cumulative hospital admissions (gray) and deaths (red) due to malaria in the children’s ward recorded from Health Management Information System data. (**B**) Comparisons of malaria case fatality before, during, and after community case management (CCM) with reactive test-and-treat (RTAT) using individual-level patient data. Patients were included if they met leukocyte and platelet criteria for severe malaria as defined by Watson et al.^15^ Overall, malaria-associated hospital mortality decreased during the CCM + RTAT period compared with pre-CCM + RTAT, with variations observed across age, sex, and village-to-hospital distance. F = female; M = male.

Pediatric hospital admissions for malaria declined during CCM + RTAT but rose again in the postintervention period (Figure 3). Among children younger than 5 years old, the average number of monthly admissions was significantly lower during the CCM + RTAT period compared with both before and after (47 versus 61 versus 79, *P* <0.001, Poisson regression adjusted for month and year). A similar trend was observed in patients ages 5 years old and older, with average monthly admissions falling during CCM + RTAT compared with the periods before and after (30 versus 39 versus 45, *P* = 0.013).

### Severe malaria and mortality.

Hospitalized children with severe malaria are described in Tables 1 and 2. There was significantly lower in-hospital mortality during CCM + RTAT than before or after (3% versus 12% versus 9%, Fisher exact *P* = 0.011). Children presented with less severe disease, indicated by higher average hemoglobin (7.0 versus 5.0 versus 5.4 g/dL, *P* <0.001) and lower prevalence of severe malarial anemia (22% versus 48% versus 44%, *P* <0.001).

**Table 1:** Characteristics of hospitalized children with severe malaria stratified by program period

Characteristic	Pre-CCM + RTAT	Intra-CCM + RTAT	Post-CCM + RTAT	*P*-Value
	*n* = 499	*n* = 107	*n* = 90	
Age (years)	2.0 (1.5–3.0)	2.0 (1.1–3.2)	2.6 (1.6–4.0)	0.13
<2 (%)	38	47	36	0.18
2 to <5 (%)	47	38	42	–
5–15 (%)	16	15	22	–
Sex (female)	51	61	47	0.09
Home village distance from hospital (km)	7.2 (2–20)	4.0 (2–15)	5.0 (2–15)	0.19
<2 (%)	21	21	25	0.12
2 to <10 (%)	32	43	42	–
10 to <20 (%)	22	18	11	–
≥20 (%)	25	18	22	–
Hemoglobin (g/dL)	5.0 (4.0–7.6)	7.0 (5.2–8.8)	5.4 (4.0–6.9)	<0.001
Leukocyte count (10^3^/*µ*L)	9.3 (6.2–13.4)	9.1 (6.3–13.3)	8.4 (6.3–11.3)	0.33
Platelet count (10^5^/*µ*L)	101 (60–161)	103 (45–152)	87 (60–138)	0.59
Length of stay (days)	3 (2–5)	3 (2–5)	3 (2–5)	0.25
Severe malaria phenotype (%)				
Severe anemia	48	22	44	<0.001
Cerebral malaria	<1	<1	<1	–
Unspecified	51	77	55	<0.001
Concomitant diagnoses (%)				
Gastroenteritis	3	5	2	0.63
Pneumonia	6	5	3	0.53
Mortality (%)	12	3	9	0.01

CCM = community case management; RTAT = reactive test-and-treat. Data are shown as percentage or median (interquartile range). *P*-values were calculated from the Pearson χ^2^ test or the Fisher exact test for dichotomous variables and from the Kruskal–Wallis test for continuous variables.

**Table 2:** Characteristics of hospitalized children with severe malaria stratified by survival

Characteristic	Survivors	Nonsurvivors	*P*-Value
	*n* = 627	*n* = 69	
Age (years)	2.1 (1.4–3.7)	2.0 (1.5–2.8)	0.10
<2 (%)	38	46	0.40
2 to <5 (%)	45	39	–
5–15 (%)	17	14	–
Sex (female)	52	54	0.80
CCM + RTAT period (%)			
Pre	70	84	0.01
Intra	17	4	–
Post	13	12	–
Home village distance from hospital (km)	6.0 (2–17)	7.2 (2–27)	0.40
<2 (%)	22	14	0.27
2 to <10 (%)	34	40	–
10 to <20 (%)	21	16	–
≥20 (%)	23	30	–
Hemoglobin (g/dL)	5.5 (4.2–8.0)	4.4 (3.7–5.8)	<0.001
Leukocyte count (10^3^/µL)	9.0 (6.2–12.5)	13.2 (7.1–16.5)	<0.001
Platelet count (10^5^/µL)	103 (63–161)	64 (44–117)	<0.001
Length of stay (days)	3 (2–5)	1 (0–2)	<0.001
Severe malaria phenotype (%)			
Severe anemia	42	57	0.02
Cerebral malaria	0	0	–
Unspecified	58	43	0.02
Concomitant diagnoses (%)			
Gastroenteritis	3	4	0.49
Pneumonia	6	6	0.99

CCM = community case management; RTAT = reactive test and treat. Data are shown as percentage or median (interquartile range). *P*-values were calculated from the Pearson χ^2^ test or the Fisher exact test where appropriate for categorical variables and from the Wilcoxon rank-sum test for continuous variables.

The odds of death after adjustment for age, sex, hemoglobin, platelet count, distance from hospital, and gastroenteritis were significantly lower during CCM + RTAT than before (odds ratio [OR]: 0.24, 95% CI: 0.07–0.82, *P* = 0.023); however, the adjusted comparison between CCM + RTAT and post-CCM + RTAT did not meet statistical significance (OR: 0.58, 95% CI: 0.13–2.7, *P* = 0.49).

### Geographic trends and subgroup analyses.

**Figure 4.**
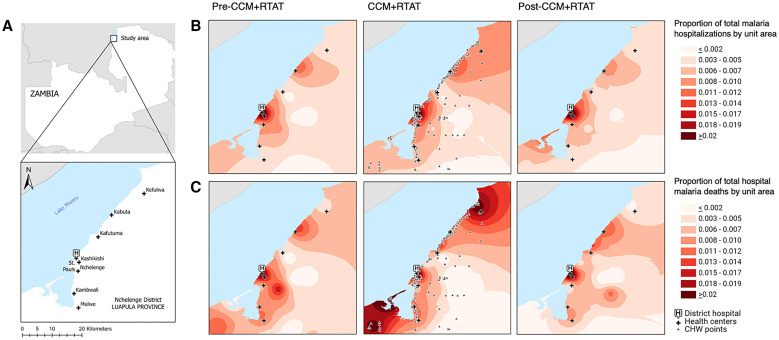
(**A**) Map of the study area showing the hospital and rural health centers. (**B**) Spatial distribution of the home villages of pediatric patients admitted with a diagnosis of malaria spanning the pre-community case management (pre-CCM) + reactive test-and-treat (RTAT) period, intra-CCM + RTAT period, and post-CCM + RTAT period. (**C**) Spatial distribution of the home villages of those patients who died of malaria. Data from the five southernmost health centers were excluded because of a boundary effect caused by two nearby hospitals located beyond the district border. CHW = community health worker.

**Figure 5.**
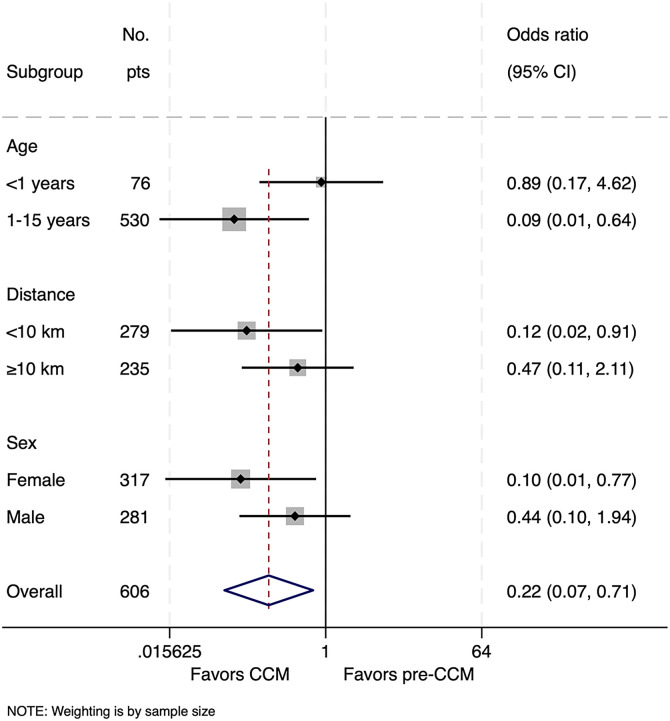
Subgroup analyses of the association of community case management (CCM) + reactive test-and-treat (RTAT) with mortality comparing the CCM + RTAT period with the pre-CCM + RTAT period. Mortality reductions were mainly seen in children 1–15 years old, children from villages closer to the hospital, and female children. Pts = patients.

Nearly all (95%) children with malaria hospitalized during the CCM + RTAT period lived within 1 km of at least one CHW. Maps showed an expansion of the area from where hospitalized children with malaria resided during the CCM + RTAT period, which reverted when CCM + RTAT was discontinued (Figure 4). On average, however, children came from villages nearer to the hospital during and after CCM + RTAT than before CCM + RTAT, although the difference was not statistically significant (Table 1), and subgroup analysis further showed that children from remote villages had no significant reduction in mortality during CCM + RTAT (Figure 5). Subgroup analysis also suggested that CCM + RTAT was associated with a greater reduction in mortality in girls compared to boys, although the CIs overlapped and the interaction term was not significant (*P* = 0.24).

## DISCUSSION

We assessed the impact of CCM + RTAT for malaria in a high-transmission setting in Zambia and identified associations between the program and increased access to care, a shift in case burden from health facilities to CHWs, reduced in-hospital mortality in children with severe malaria possibly because of earlier referral of cases, and a wider geographic catchment. These findings align with two key program objectives: reducing the patient loads of clinic staff, who can then turn their efforts toward higher acuity patients, and geographically expanding access to care.

Previous studies showed varying degrees of impact of CCM on malaria morbidity and mortality (without RTAT). In Uganda and Ghana, CCM led to more frequent treatment of malaria in children by CHWs.^4^^,^^6^ A 4-year impact evaluation of integrated CCM (iCCM) rolled out at six sites in the Democratic Republic of the Congo, Mozambique, Malawi, Niger, and Nigeria estimated that iCCM reduced mortality in children younger than 5 years old by 10%, although the impact was mainly attributed to treatment of pneumonia and diarrhea.^17^ The study also identified a perennial challenge to programs: in Mozambique, no impact was seen because of widespread stockouts of essential medicine.^17^

Coverage of indoor residual spraying was low in the preintervention period and increased over time, but it did not account for any of the differences in malaria metrics across the periods. This is consistent with our prior work that showed minimal to no impact of indoor residual spraying on parasite prevalence.^18^

The significantly reduced in-hospital mortality in children with severe malaria observed during CCM + RTAT could be explained by improvements in early diagnosis and referral, a tenet of CCM. We observed higher mean hemoglobin concentration and lower frequency of severe anemia compared with either before or after CCM + RTAT, consistent with earlier referral and less advanced disease. The reduced prevalence of severe malarial anemia during CCM + RTAT likely decreased reliance on blood transfusions, an important outcome given frequent blood stockouts in the hospital, which are associated with higher mortality in children who cannot receive necessary transfusions.^12^^,^^19^ The incorporation of RTAT into the CCM program could explain why the impact on malaria mortality was greater than seen in prior evaluations of CCM (e.g., by detecting cases that otherwise would have been missed even with effective CCM). However, RTAT in high malaria transmission settings is labor and resource intensive and prohibitively costly to sustain.

Although we found evidence that the program reached children from more remote villages, these children suffered similar mortality during the program than before or after the program. This underlines the need for prereferral treatment and better emergency transportation. The rural access index for Nchelenge District, which measures the proportion of a population with adequate access to road infrastructure, is 16%, and the availability of vehicles is poor.^20^

The subgroup analysis suggested that girls may have benefitted from CCM + RTAT more than boys. If this were true, both biological and behavioral factors could explain why. If disease progression differs based on biologic sex differences—some studies suggest that progression is more rapid in girls^21^^,^^22^—then earlier diagnosis and referral could have a disproportionate impact across sexes. Behavioral or other psychosocial differences in health care seeking or how disease in children comes to the attention of parents are other potential explanations that could be explored in qualitative studies.

It is impossible to tell from our data whether the increase in mortality seen after CCM + RTAT ended was solely because of a return to the pre-CCM + RTAT state or whether there was a contribution of “rebound malaria,” where clinical immunity wanes on account of a successful intervention, rendering the population more vulnerable to severe outcomes when the intervention is then withdrawn.^23^

Strengths of this study include three independent data sources with large sample sizes that showed concordant findings, sufficient duration of the intervention to capture annual fluctuations in malaria, and the natural crossover design afforded by the program rollout and subsequent rollback. An inherent limitation of this study is that CCM was implemented in combination with RTAT, limiting direct comparisons with CCM-only interventions from other studies. Another limitation is reliance on indirect indicators within retrospective data; for the community cohort, we did not directly measure health care contacts with CHWs, relying instead on existing survey instruments that did not distinguish whether treatment was received from a CHW or a health facility. Similarly, patient data were retrospective, and we could not identify whether a given patient had contact with a CHW or not. The postprogram period coincided with the first year of the coronavirus pandemic, but we did not detect any drop-off in health seeking for malaria or other apparent influences of the pandemic.

## CONCLUSION

The results of this impact assessment present a conundrum. RTAT is contraindicated in areas of high malaria transmission because of the avid consumption of malaria commodities, which threatens to deprive patients of needed diagnostic tests and drugs when they are in short supply. Yet, we found that CCM + RTAT was associated with a significant reduction in malaria mortality. Although we do not advocate for the further study or adoption of RTAT in high-transmission settings, these results might justifiably kindle policy discussions around other case detection-based approaches to malaria control in holoendemic areas, which are rarely discussed in this context.

## References

[1] World Health Organization, 2023. World Malaria Report. Geneva, Switzerland: WHO Global Malaria Programme.

[2] Amouzou A Morris S Moulton LH Mukanga D, 2014. Assessing the impact of integrated community case management (iCCM) programs on child mortality: Review of early results and lessons learned in sub-Saharan Africa. J Glob Health 4: 020411. 25520801 10.7189/jogh.04.020411 PMC4267100

[3] World Health Organization, 2022. WHO Guidelines for Malaria. Geneva, Switzerland: WHO.

[4] Chinbuah AM Gyapong JO Pagnoni F Wellington EK Gyapong M, 2006. Feasibility and acceptability of the use of artemether‐lumefantrine in the home management of uncomplicated malaria in children 6–59 months old in Ghana. Trop Med Int Health 11: 1003–1016. 16827701 10.1111/j.1365-3156.2006.01654.x

[5] Hamer DH Brooks ET Semrau K Pilingana P MacLeod WB Siazeele K Sabin LL Thea DM Yeboah-Antwi K, 2012. Quality and safety of integrated community case management of malaria using rapid diagnostic tests and pneumonia by community health workers. Pathog Glob Health 106: 32–39. 22595272 10.1179/1364859411Y.0000000042 PMC4001509

[6] Källander K Tomson G Nsabagasani X Sabiiti JN Pariyo G Peterson S, 2006. Can community health workers and caretakers recognise pneumonia in children? Experiences from western Uganda. Trans R Soc Trop Med Hyg 100: 956–963. 16455119 10.1016/j.trstmh.2005.11.004

[7] Zambia Ministry of Health, 2022. National Malaria Elimination Strategic Plan 2022–2026. Lusaka, Zambia: Zambia National Malaria Elimination Program.

[8] Ippolito MM , 2018. Risk factors for mortality in children hospitalized with severe malaria in northern Zambia: A retrospective case-control study. Am J Trop Med Hyg 98: 1699–1704. 29692306 10.4269/ajtmh.17-1017 PMC6086172

[9] National Malaria Control Centre, 2017. National Malaria Elimination Strategic Plan 2017–2021 (Zambia). Lusaka, Zambia: National Malaria Control Centre.

[10] Zambian Ministry of Health, Zambia National Malaria Elimination Programme, PATH, United States President’s Malaria Initiative, United Nations Children’s Fund, World Health Organization, 2021. Zambia Malaria Indicator Survey 2021. Lusaka, Zambia: Zambia National Malaria Elimination Programme.

[11] Ippolito MM , Southern and Central Africa International Center of Excellence for Malaria Research, 2022. Scientific findings of the Southern and Central Africa International Center of Excellence for Malaria Research: Ten years of malaria control impact assessments in hypo-, meso-, and holoendemic transmission zones in Zambia and Zimbabwe. Am J Trop Med Hyg 107(4 Suppl): 55–67. 36228903 10.4269/ajtmh.21-1287 PMC9662223

[12] Ippolito MM , Southern and Central Africa International Centers of Excellence for Malaria Research, 2022. Whole blood transfusion for severe malarial anemia in a high *Plasmodium falciparum* transmission setting. Clin Infect Dis 75: 1893–1902. 35439307 10.1093/cid/ciac304 PMC10200327

[13] van Buuren S, 2007. Multiple imputation of discrete and continuous data by fully conditional specification. Stat Methods Med Res 16: 219–242. 17621469 10.1177/0962280206074463

[14] Ippolito MM Robinson ML, 2022. Reducing misclassification bias in severe malaria research. Cell Rep Med 3: 100789. 36260989 10.1016/j.xcrm.2022.100789 PMC9589118

[15] Watson JA , 2021. Improving statistical power in severe malaria genetic association studies by augmenting phenotypic precision. Elife 10: e69698. 34225842 10.7554/eLife.69698 PMC8315799

[16] Hauser M , 2023. Malaria in refugee children resettled to a holoendemic area of sub-Saharan Africa. Clin Infect Dis 76: e1104–e1113. 35640824 10.1093/cid/ciac417 PMC10169438

[17] Prosnitz D Herrera S Coelho H Moonzwe Davis L Zalisk K Yourkavitch J, 2019. Evidence of impact: iCCM as a strategy to save lives of children under five. J Glob Health 9: 010801. 31263547 10.7189/jogh.09.010801 PMC6594661

[18] Hast MA , 2019. The impact of 3 years of targeted indoor residual spraying with pirimiphos-methyl on malaria parasite prevalence in a high-transmission area of northern Zambia. Am J Epidemiol 188: 2120–2130. 31062839 10.1093/aje/kwz107 PMC7212407

[19] Kabuya J-BB , 2024. Supplementing routine hospital surveillance of malaria to capture excess mortality and epidemiological trends: A five-year observational study. Frontiers in Malaria 2: 2024.1340276. 10.3389/fmala.2024.1340276 PMC12175441 40535552

[20] World Bank Group, 2016. Measuring Rural Access Using New Technologies. Washington, DC: World Bank Group.

[21] Briggs J , 2020. Sex-based differences in clearance of chronic *Plasmodium falciparum* infection. Elife 9: e59872. 33107430 10.7554/eLife.59872 PMC7591246

[22] Okiring J , 2022. Gender difference in the incidence of malaria diagnosed at public health facilities in Uganda. Malar J 21: 22. 35062952 10.1186/s12936-022-04046-4 PMC8778495

[23] Greenwood B Zongo I Dicko A Chandramohan D Snow RW Ockenhouse C, 2022. Resurgent and delayed malaria. Malar J 21: 77. 35264158 10.1186/s12936-022-04098-6 PMC8905818

